# Supplementary material for: EAU Biochemical Recurrence Risk Classification and PSA Kinetics Have No Value for Patient Selection in PSMA-Radioguided Surgery (PSMA-RGS) for Oligorecurrent Prostate Cancer
Source: Cancers (Basel). 2023 Oct 16;15(20):5008. doi: 10.3390/cancers15205008 (PMC10605818; doi:10.3390/cancers15205008)
Supplement: Supplementary file 1 [file cancers-15-05008-s001.zip › cancers-2631232-supplementary.pdf]

Supplementary Material

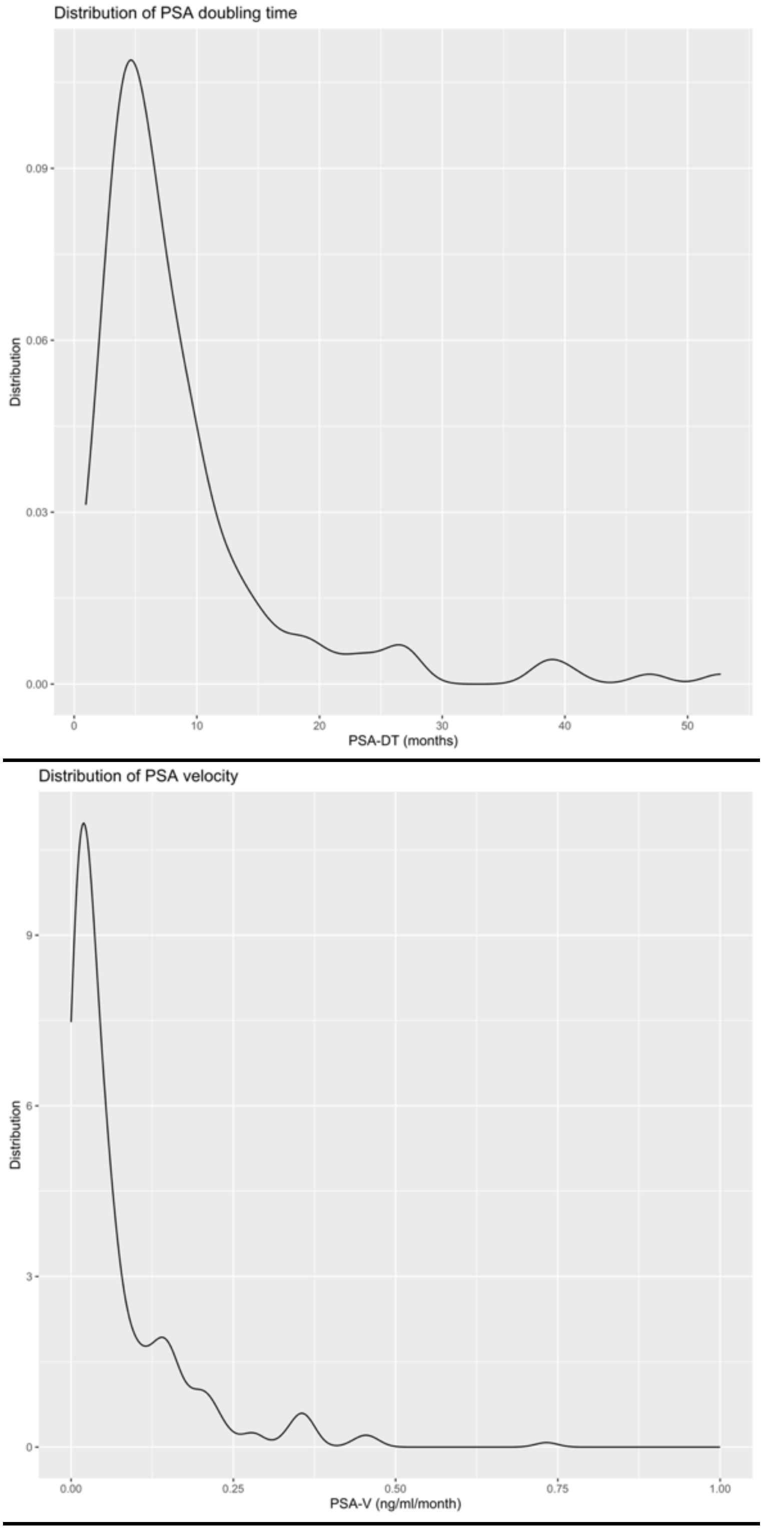

Figure S1. Distribution of PSA-DT and PSA-V within the cohort as density function.
